# Lysine-Specific Demethylase 1A as a Promising Target in Acute Myeloid Leukemia

**DOI:** 10.3389/fonc.2018.00255

**Published:** 2018-07-19

**Authors:** Daniela Magliulo, Rosa Bernardi, Samantha Messina

**Affiliations:** ^1^Vita-Salute San Raffaele University, Milan, Italy; ^2^Laboratory of Preclinical Models of Cancers, Division of Experimental Oncology, San Raffaele Scientific Institute, Milan, Italy; ^3^Department of Human Sciences, Society and Health, University of Cassino and Southern Lazio, Cassino, Italy

**Keywords:** LSD1, epigenetics, leukemia stem cells, acute myeloid leukemia, LSD1 inhibitors

## Abstract

Acute myeloid leukemia (AML) is a heterogeneous hematopoietic malignancy characterized by the accumulation of incompletely differentiated progenitor cells (blasts) in the bone marrow and blood, and by suppression of normal hematopoiesis. It has recently become apparent that the AML genome is characterized by recurrent mutations and dysregulations in epigenetic regulators. These mutations frequently occur before the onset of full blown leukemia, at the pre-leukemic phase, and persist in residual disease that remains after therapeutic intervention, thus suggesting that targeting the AML epigenome may help to eradicate minimal residual disease and prevent relapse. Within the AML epigenome, lysine-specific demethylase 1 A (*LSD1*) is a histone demethylase that is found frequently overexpressed, albeit not mutated, in AML. *LSD1* is a required constituent of critical transcription repressor complexes like CoREST and nucleosome remodeling and deacetylase (NuRD), and abrogation of *LSD1* expression results in impaired self-renewal and proliferation, and increased differentiation and apoptosis in AML models and primary cells, particularly in AMLs with MLL- and AML1-rearrangements, or erythroid and megakaryoblastic differentiation block. On this basis, a number of LSD1 inhibitors have been developed in the past decade, and few of them are currently being tested in clinical trials for patients with AML, along with other malignancies. To date, the most promising application of this therapeutic strategy appears to be combination therapy of LSD1 inhibitors with all-trans retinoic acid (ATRA) to reactivate myeloid differentiation in cells that are not spontaneously susceptible to ATRA treatment. In this review, we provide an overview of *LSD1* function in normal hematopoiesis and leukemia, and of the current clinical application of LSD1 inhibitors for the treatment of patients with AML.

## Introduction

Acute myeloid leukemia (AML) is a clonal disorder of myeloid progenitors characterized by vast clinical and biological heterogeneity (1). Recent work on the genetic and molecular characterization of AML has revealed that this disease is often associated with dysregulated epigenetic mechanisms, caused by somatic mutations in genes playing key functions in epigenetic regulation as well as more widespread epigenetic modifications caused by altered activity of epigenetic factors coopted by mutated transcriptional regulators (2).

Epigenetic regulation of gene expression defines inheritable regulatory mechanisms that do not affect the DNA sequence. Beside DNA modifications, mainly methylation, epigenetic mechanisms include RNA-associated gene silencing and histone modifications. Histone proteins (H2A, H2B, H3, and H4) assemble with DNA to form the basic unit of chromatin, the nucleosome (3), and large families of enzymes have been shown to promote post-translational modifications (PTMs) of histones resulting in fine regulation of gene expression (4, 5). Histone modifications include phosphorylation, acetylation, methylation, ubiquitination, SUMOylation, and GlcNAcylation, and different proteins have been described to add (writers), remove (erasers), and recognize (readers) histone PTMs, thus regulating transcriptional responses in many cell types (6).

In this review, we will focus on the epigenetic eraser lysine-specific demethylase 1A (LSD1), a lysine demethylase acting on histones H3K4me1/2 and H3K9me1/2 (7). The discovery of LSD1 in 2004 demonstrated for the first time that histone methylation is a reversible reaction (8). After an initial characterization of the function of LSD1 in embryonic development, a number of studies have described LSD1 as implicated in tumorigenesis, and found it overexpressed in many solid tumors, where it impairs differentiation, promotes proliferation, cell motility and invasiveness, and associates with poor prognosis (9–13). In the context of blood neoplasms, LSD1 was found overexpressed in about 60% of acute myeloid leukemia cases (14, 15), where recent work has suggested that it contributes to leukemia development and propagation by imposing a myeloid maturation arrest and promoting proliferation of myeloid progenitors (16, 17).

Acute myeloid leukemia patients are currently treated with intensive chemotherapy regimens (except elderly or frail patients) and stem cell transplantation protocols. However, the majority of AML patients still succumb to this disease because of refractoriness to therapy or relapse, and new therapeutic approaches for AML are an urgent clinical need (2). In the last decade, several epigenetic modulators have been developed and tested in preclinical and clinical studies. Within this category, LSD1 inhibitors constitute a promising epigenetic approach to treat AML, and currently a number of clinical trials are aiming to treat refractory AML by blocking LSD1 activity (15, 18).

In this review, we will discuss the role of LSD1 in normal hematopoiesis and in AML, and describe novel therapeutic approaches targeting this molecule for AML treatment.

## Structure and Functions of LSD1

The family of human histone demethylases includes up to 21 different lysine-specific enzymes that modify chromatin to promote or repress gene transcription in a residue-selective manner. The larger Jumonji C family (JMJCs) depends on α-ketoglutarate and Fe^2+^ and acts on tri-, di-, or mono-methylated lysine residues, while the smaller LSD family consists of only two members (LSD1 and LSD2) (7). LSD1, also known as KDM1A, AOF2, or BHC110, was the first identified histone demethylase (8). It is a flavine-adenine dinucleotide (FAD)-dependent amine oxidase (AO) that demethylates di- and mono-methyl groups on H3K4 and H3K9 and few non-histone targets (such as DNMT1, p53, E2F1, and HIF-1α) (19–23).

**Figure 1:**
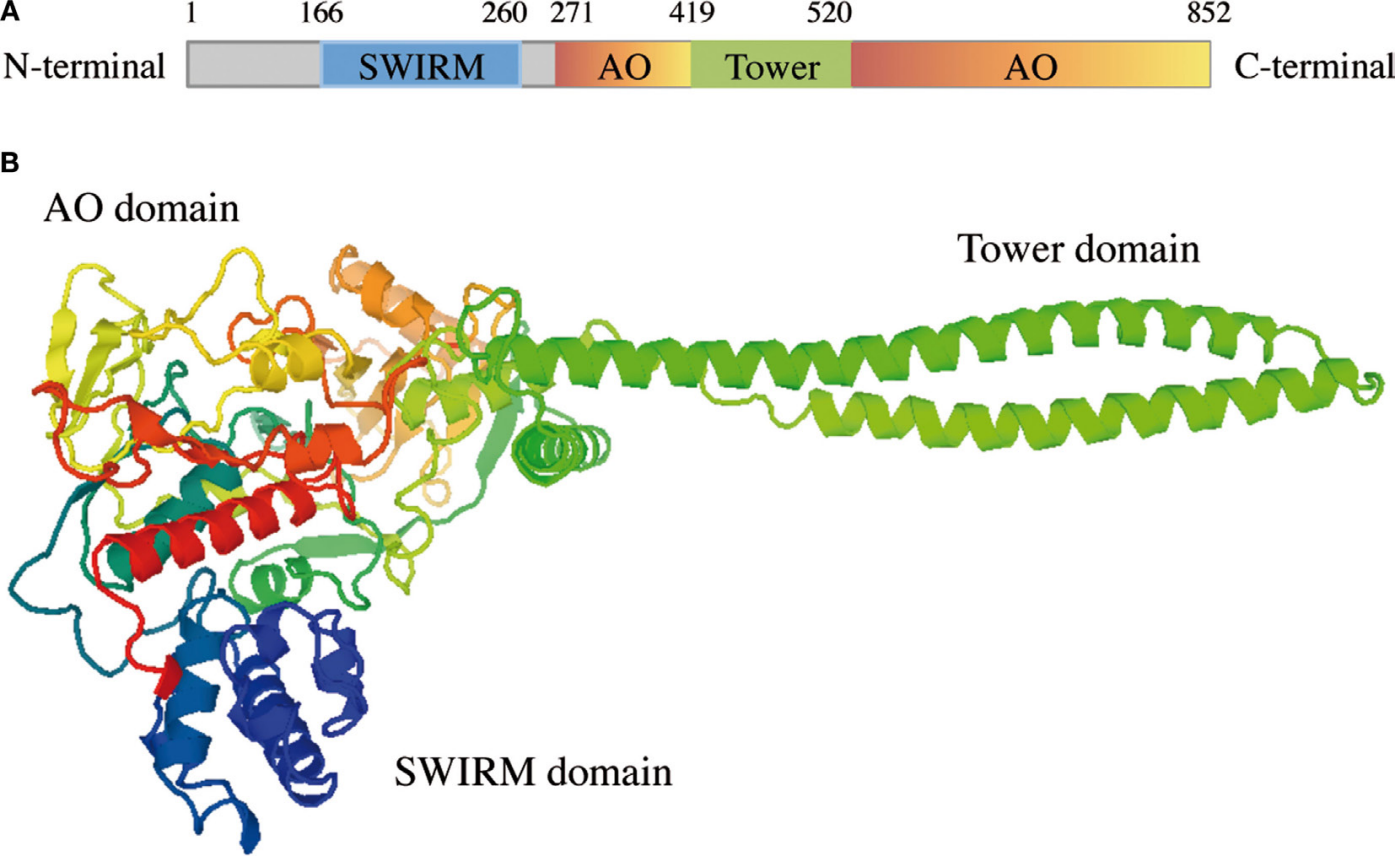
LSD1 protein structure. **(A)** Schematic illustration of LSD1 domains. **(B)** Tridimensional structure of LSD1; domains are colored as in **(A)**. The image was obtained from SWISS-MODEL protein structure homology-modeling server (25).

LSD1 has three structural domains that regulate its enzymatic activity and binding to several proteins. From the N-terminus this enzyme consists of a SWIRM domain (named after the Swi3p, Rsc8p, and Moira proteins in which it was first identified), a Tower domain and a C-terminal FAD-dependent AO domain that surrounds the Tower domain and consists of two different lobes (Figure 1A) (24).

A disordered area of 170 residues, which can be post-translationally modified and promotes protein–protein interactions, characterizes the N-terminal extremity (26). The SWIRM domain follows this region and is shaped as a small alpha-helix that lacks the DNA-binding ability typical of other SWIRM domains but maintains its protein–protein interaction ability (27, 28). The AO domain is the catalytic region of LSD1 and consists of two lobes, the FAD-binding site and the substrate recognition site, shaped in a more open conformation than any other FAD-dependent monoamine oxidase (29, 30). In the tertiary structure of LSD1, the second lobe of the AO domain is in close proximity to the SWIRM domain, thus forming a hydrophobic groove that allows LDS1 to accommodate a large portion of histone H3 tail, sense epigenetic marks, and modify chromatin accessibility thought its demethylating activity (Figure 1B) (31). Finally, the Tower domain consists of two antiparallel helices that in the tertiary structure of LSD1 protrude from the AO domain and act as a platform for binding to RCOR1, a member of the CoREST transcriptional repressor complex (32, 33).

**Figure 2:**
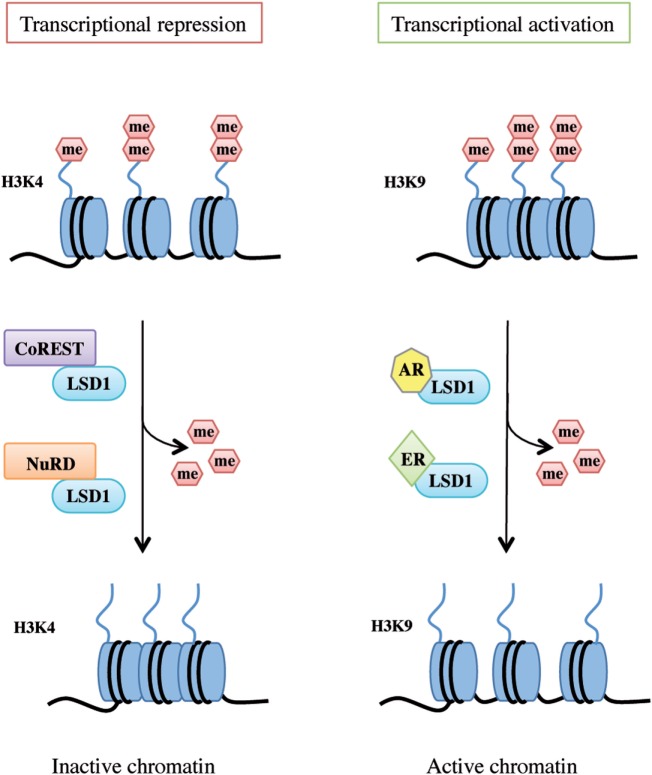
Lysine-specific demethylase 1A (LSD1) dual functions as transcriptional repressor and activator. LSD1 regulates chromatin accessibility through its demethylating activity on histone H3 Lys4 and Lys9 residues. On the left, LSD1 binds to the CoREST or nucleosome remodeling and deacetylase repressive complex thus demethylating mono- and dimethyl-group on histone H3K4 and allowing genes transcriptional repression. On the right, following androgen receptor or estrogen receptor binding, LSD1 promotes transcriptional activation by demethylating mono- and dimethyl-group on histone H3K9.

LSD1 exerts dual functions by acting as a transcription co-repressor or a co-activator through H3K4 or H3K9 demethylation, respectively (Figure 2).

### LSD1 as a Transcriptional Co-Repressor: H3K4 Demethylation

Histone 3 lysine 4 methylation is a mark of active transcription. In particular, mono-methylated H3K4 denotes primed enhancer elements, di-methylated H3K4 active enhancers and promoters, and tri-methylated H3K4 transcriptionally active promoters (34). LSD1 binds to several proteins and takes part in different complexes to promote H3K4 demethylation and shape chromatin into a repressive configuration, such as the CoREST transcriptional repressor complex and the Mi-2/nucleosome remodeling and deacetylase (NuRD) complex (Figure 2, left panel).

The CoREST complex consists of numerous proteins, including LSD1, RCOR1, HDAC1/2, ZNF217, PHF21A, and HMG20B (35). The interaction of LSD1 with RCOR1 allows the formation of an H3-tail binding site that recognizes a 20 amino acids portion of the histone H3 N-terminal region (36, 37). HDAC1/2 is essential to remove local acetylation marks on histone H3, an event that promotes LSD1 demethylation activity (38, 39). PHF21A, the first reader of histone modifications to be discovered, senses unmethylated H3K4 and is required to stabilize LSD1 on its target regions and mediate demethylation of the surrounding nucleosomes (40). Most of the other members of the complex are much less characterized and need to be further studied.

The NuRD repressor complex is also composed of several proteins, including Mi-2, MTA, RBBP, MBD2/3, and HDAC1/2. As for the CoREST complex, the NuRD complex combines the deacetylation of histone H3 and the demethylation of histone H3K4 to repress transcription. In addition, targets of the LSD1/NuRD complex are often genes involved in cell signaling pathways regulating cell proliferation, survival, and epithelial-to-mesenchymal transition in specific cell-contexts (41, 42).

### LSD1 as a Transcriptional Co-Activator: H3K9 Demethylation

Upon specific stimulation, LSD1 can also act as a transcriptional co-activator. For example, following androgen receptor pathway activation, LSD1 demethylates mono- and dimethyl-H3K9 repressive marks and promotes transcription of androgen target genes in prostate cancer cells (23, 28). Moreover, upon stimulation of estrogen receptor (ER) signaling, LSD1 acts on dimethyl-H3K9 sites on enhancers and promoters of estrogen-induced genes to drive their expression (Figure 2, right panel) (43). Interestingly, a recent study by Bennesch et al. demonstrated that recruitment of the CoREST complex is essential for LSD1 function in the activation of the ER pathway in breast cancer cells, thus indicating that repressive complexes may perform gene activation functions in specific cell types, although further studies are needed to fully elucidate the molecular intersection of the ER pathway and CoREST complex activity (44).

## LSD1 Role in Hematopoiesis

Since its discovery in 2004, *LSD1* has been increasingly described as a gene regulating crucial cellular and organismal processes, ranging from embryonic development to adult tissue homeostasis and cellular differentiation (8, 33). First, *LSD1* is highly expressed in embryonic stem cells, while being downregulated during differentiation, and *in vivo* genetic deletion and loss-of-function gene trapping of *LSD1* causes impaired growth and a developmental arrest at an early stage of embryogenesis, due to the regulation of key developmental factors by LSD1 (45, 46).

Within the hematopoietic system, many studies have demonstrated that *LSD1* is a critical regulator of normal hematopoiesis and leukemogenesis. *LSD1* has a crucial role in regulating hematopoietic stem cells (HSCs) maturation and differentiation at different stages of development.

### Role of LSD1 in Embryonic Hematopoiesis

**Figure 3:**
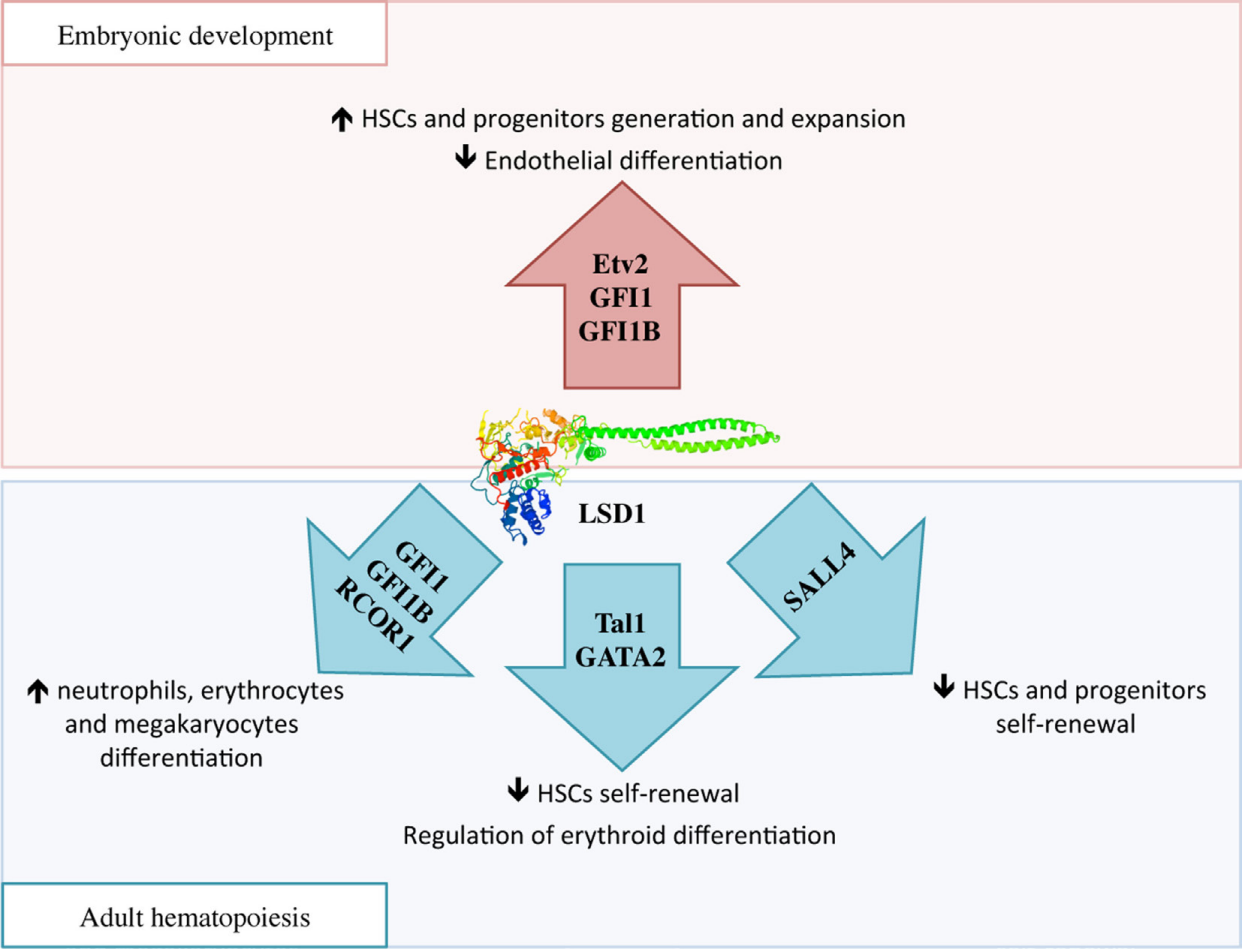
The complex function of lysine-specific demethylase 1A (LSD1) in hematopoiesis. During embryonic development, LSD1 promotes expansion and maturation of hematopoietic stem and progenitor cells by regulating *Evt2* expression and downregulating genes involved in endothelial commitment through binding to GFI1 and GFI1B. In adult hematopoiesis, LSD1 limits hematopoietic stem cells self-renewal through Sal-like protein 4 and Tal1 binding; at the same time it promotes hematopoietic differentiation toward neutrophils, erythrocytes, and megakaryocytes through specific binding to RCOR1/CoREST and GFI1 and GFI1B. Moreover, LSD1–Tal1 complex regulates erythroid differentiation through GATA2 binding and expression of the *Gata1* gene at different stages of hematopoiesis.

During embryonic development, HSCs are generated in two waves: in primitive hematopoiesis, hematopoietic and endothelial cells share a common progenitor called the hemangioblast, while in the definitive wave of hematopoiesis, HSCs develop from the hemogenic endothelium through a transdifferentiation process known as endothelial-to-hematopoietic transition (EHT) (47). In the hemangioblast, LSD1 downregulates *Etv2*, an important inducer of endothelial differentiation, thus promoting the expansion of hematopoietic progenitors (Figure 3) (48). Later, essential molecules in the generation of HSCs are two transcriptional repressors, GFI1 and GFI1B, which are highly expressed during EHT. These proteins display at the N-terminal region a 20 amino acids SNAG (Snail/GFI1) domain that resembles the histone H3 tail. This region is methylated by the specific lysine-methylase SMYD2 thus promoting LSD1 binding (49, 50). GFI1/GFI1B–LSD1 interaction is involved in the downregulation of genes associated with the endothelial program and development of the cardiovascular system, and in the activation of genes associated with HSCs generation and maturation (Figure 3) (51).

### Role of LSD1 in Adult Hematopoiesis

*In vivo* experiments of *LSD1* conditional deletion in fetal (VavCre) and adult (Mx1Cre) hematopoietic cells show that LSD1 loss causes defects in long-term hematopoietic stem cells self-renewal. At the same time, however, LSD1-deficiency causes severe pancytopenia and impaired HSCs differentiation toward immature progenitors and mature granulocytes and erythrocytes (52). These latest results were corroborated by another study that used an *in vivo* stable conditional knockdown model of LSD1 (shLSD1), where it was shown that LSD1 is required for terminal granulocytic, erythroid, and megakaryocytic maturation (53).

Mechanistically, several studies are suggesting that LSD1 exerts negative regulation of HSCs self-renewal by functionally interacting with critical transcription factors involved in stem cell maintenance, and repressing stem and progenitor gene expression programs (52). For example, in HSCs and hematopoietic progenitors, LSD1 co-localizes in the nucleus with Sal-like protein 4 (SALL4), a zing-finger transcription factor involved in promoting self-renewal and expansion of HSCs. LSD1 negatively regulates both SALL4 itself, as its mRNA level was found increased upon LSD1 silencing, as well as expression of its target genes by acting on H3K4 methylation at their promoters (Figure 3) (54).

During hematopoietic differentiation, the regulatory functions of LSD1 are mediated by its interactions with RCOR proteins of the CoREST complex and transcription factors like Tal1 and GFI1 (Figure 3) (15). In undifferentiated cells, Tal1 (also known as Scl1) is phosphorylated at serine 172 in the LSD1-interacting domain. This modification allows LSD1–Tal1 interaction and LSD1 binding to promoters of Tal1-target genes involved in HSCs self-renewal, thus repressing their transcription through the regulation of H3K4 methylation status (55, 56). Recently, it has been shown that LSD1–Tal1 complexes associate with the GATA2 transcription factor during erythropoietic differentiation. At early stages of erythropoiesis, this complex downregulates *Gata1* gene expression, while later in the differentiation, LSD1 is released from GATA2 and promotes expression of GATA1, an important driver of erythroid differentiation (57). In addition, LSD1 and RCOR1 also associate with the GFI1 and GFI1B transcription factors, which critically regulate differentiation to neutrophils, erythrocytes, and megakaryocytes, to repress expression of their target genes (58). In conclusion, it is believed that hematopoietic differentiation defects observed in LSD1-deficient cells may be caused by the untimely and uncontrolled activity of these transcription factors (15).

Considering the key role of LSD1 in hematopoiesis, its regulation becomes fundamental in pathological conditions like emergency hematopoiesis, a condition in which HSCs respond to severe infections by expanding the production of immune cells. Indeed, upon viral or bacterial infections, TNFα and IL1β inflammatory cytokines suppress LSD1 activity through miRNA-driven mechanism and induce HSCs proliferation and differentiation (59).

## Regulation and Function of LSD1 in AML

Unlike other epigenetic regulators like the histone methyltransferase MLL1, LSD1 has not been found mutated in AML, but it is highly expressed in leukemic blasts in about 60% of AML patients as well as in other hematological malignancies (14). LSD1 overexpression does not appear to characterize distinct AML subtypes with specific genetic modifications; however, LSD1 is one of the genes with highest expression in leukemia stem cells (LSCs) (60), thus suggesting that it may play important functions in regulating leukemia maintenance and relapse. In line with this hypothesis, LSD1 was described as a critical regulator of LSCs potential in a mouse model of MLL-AF9-driven AML (16), by binding to genomic loci controlled by MLL–AF9 and promoting the expression of oncogenic gene expression signatures while inhibiting differentiation and apoptosis. Moreover, LSD1 knockdown or inhibition impaired the clonogenic and repopulating potential of AML LSCs, while sparing normal hematopoietic stem cells (16), thus indicating that LSD1 inhibition could be exploited as a safe strategy for targeting AML, especially AML repopulating cells.

More recently, two studies have confirmed that LSD1 plays critical functions in AML, by acting as a positive regulator of proliferation and self-renewal, and negatively impacting cell differentiation programs. Fang et al. showed that shRNA-mediated inhibition of LSD1 in AML cells, contrary to what had been previously described in the normal hematopoietic system, causes a remarkable transcriptional activation of myeloid lineage genes (CD11b/ITGAM and CD86), along with a reduction of AML cell proliferation and clonogenic ability. Chromatin immunoprecipitation analyses revealed that LSD1 silencing is accompanied by a specific increase of H3K4me2 and H3K4me3 modifications specifically at the promoter regions of CD11b and CD86, whereas global H3K4me2 levels remain constant, as previously reported (61). Cusan et al. performed a comparative assessment of chromatin dynamics during the treatment of MLL-AF9-driven murine leukemia and MLL-rearranged patient-derived xenografts with differentiation-inducing epigenetic therapies targeting LSD1 and DOT1L. Intriguingly, while the LSD1 inhibiting agent caused global gains in chromatin accessibility, treatment with the DOT1L inhibitor EPZ4777 caused an opposite phenotype. Moreover, diminished expression of two transcription factors that control differentiation-related genes (C/EBPα and PU.1) generated resistance to LSD1 inhibition, thus suggesting that pharmacological inhibition of LSD1 may represent a unique path to overcome the differentiation block of AML (62).

Although these studies consistently suggest that LSD1 expression is important for AML development and/or maintenance, particularly in the presence of MLL mutations, transgenic mice overexpressing LSD1 in hematopoietic stem and progenitor cells showed increased stem cells self-renewal through upregulation of HOXA, but did not develop overt leukemia (63). These observations, coherently with LSD1 not being mutated in AML, indicate that LSD1 overexpression may be a secondary event during acute myeloid leukemogenic. Nonetheless, neoplastic cells that overexpress LSD1 appear to critically depend on its gene regulation activity, and inhibiting LSD1 functions is being increasingly investigated as a new therapeutic approach.

## LSD1 Inhibitors in Clinical Development in AML

One important rationale to develop LSD1 inhibitors for AML treatment came from preclinical studies, where LSD1 inhibition was coupled with all-trans retinoic acid (ATRA) with the aim of de-repressing myeloid differentiation genes and sensitizing non-APL AMLs to ATRA-induced differentiation, thus expanding the extraordinary benefit of differentiation therapy beyond the boundaries of APL (17). In the same year, it was observed that pharmacological inhibition or genetic knockdown of LSD1 induced exhaustion of LSCs in a mouse model of MLL-AF9-driven AML (16). Molecular characterization of LSD1 inhibition revealed that in LSCs LSD1 crucially regulates the expression of oncogenic MLL–AF9 target genes by modifying H3K4 methylation at their promoters (16).

These studies provided the first clues that LSD1 inhibition, either as monotherapy or in combination with ATRA, could provide a new promising approach for treating AML. Since then, in the last decade several reversible and irreversible LSD1 inhibitors have been developed and tested in preclinical AML models, with some of them entering clinical investigation (Table 1) (15). Overall these compounds appear to exert promising anti-leukemic functions primarily by altering stem cell programs, inhibiting proliferation, and restoring myeloid differentiation in AML cells, with increased efficacy for leukemia subtypes carrying MLL and AML1 rearrangements, NPM1 mutations, and erythroid and megakaryoblastic differentiation block (15). These effects are markedly enhanced in combination with ATRA or histone deacetylase inhibitors (HDACs) with little toxicity (64).

**Table 1:** LSD1 inhibitors currently tested in clinical trials for acute myeloid leukemia (AML).

Study	Phase	Trial number	Disease(s)	Drug(s)
A phase I study of pharmacokinetics and safety of ORY1001	I/II	EudraCT 2013-002447-29	Relapsed or refractory AML	ORY1001 (RG6016) (Oryzon Genomics Barcelona, Spain)
Phase 1 study of IMG7289 with or without all-trans retinoic acid (ATRA)	I	NCT02842827	AML and myelodysplastic syndromes	IMG7289 (Imago Biosciences), ATRA
Phase 1 study of TCP and ATRA	I	NTC02273102	AML and myelodysplastic syndromes	Tranylcypromine (TCP) Tretinoin (ATRA)
Phase I/II trial of TCP and ATRA	I/II	NCT02261779	Relapsed or Refractory AML	Tranylcypromine (TCP) Tretinoin (ATRA)

### Irreversible LSD1 Inhibitors

Most inhibitors of LSD1 have been designed by modifying the structure of tranylcypromine (TCP), an antidepressant originally developed to target the monoamine oxidases A and B (MAO-A and MAO-B), which are structurally related to LSD1. Upon the initial description of TCP as an irreversible inhibitor of LSD1 (65), many TCP derivatives with increased selectiveness over MAOs and/or decreased toxicity toward normal erythrocytes have been generated, and a handful of them is currently tested in clinical trials for AML (see Table 1) as well as other neoplastic diseases (15). In addition, reversible LSD1 inhibitors have been generated more recently (15). In general terms, while irreversible inhibitors have long-lasting effects on the target of interest but can also show long-lasting off-target effects if the compound is non-specific, reversible inhibitors may provide a safer profile. Due to the novelty of this targeted strategy however, the specific and side effects of different LSD1 inhibitors in patients are only beginning to be characterized, and the benefit of specific strategies will emerge soon.

The most potent and selective LSD1 inhibitor to date is ORY-1001 (PubChem CID: 71664305), designed by Oryzon Genomics from the structure of TCP. A complete *in vitro* and preclinical characterization of ORY-1001 has been recently provided, with some preliminary data from an ongoing clinical trial (66). ORY-1001 induces cell differentiation followed by reduction of cell growth and clonogenicity particularly in AML cells carrying MLL translocations or belonging to the M4 and M5 FAB subgroups (66). In addition, the authors performed a molecular characterization of gene expression and H3K4 methylation profiles, showing that a number of genes are regulated upon LSD1 inhibition, many involved in myeloid differentiation, and changes in H3K4 methylation explain only partially gene expression modifications. Nonetheless, a gene signature of LSD1 inhibition was identified in cell lines and validated in patients, thus indicating that treatment efficacy could be easily monitored in ongoing trials. Finally, the authors confirmed that LSD1 inhibition synergizes with standard of care and experimental treatments like ATRA, ARA-C, FLT3 inhibition, and other epigenetic inhibitors (66).

Other irreversible LSD1 inhibitors tested in phase I clinical trials in AML patients are GSK2879552 (GlaxoSmithKline) and IMG7289 (Imago Biosciences). GSK2879552 was identified as a potent, selective, orally bioavailable small molecule inhibitor of LSD1 in 2015 (67). However, despite showing a remarkable antiproliferative activity in both AML and small cell lung carcinoma cell lines (67), a phase I dose escalation study of GSK2879552 in subjects with AML has recently stopped as the risk benefit in relapsed refractory AML does not favor continuation of the study (CT identifier: NCT02177812). In contrast, IMG7289 is still under investigation both as single agent and in combination with ATRA in patients with AML and myelodysplastic syndrome (CT identifier: NCT02842827). This compound shows promising therapeutic efficacy by causing global gain in chromatin accessibility for PU.1 and C/EBPα transcription factors, coinciding with the induction of myeloid differentiation in MLL-rearranged AMLs (62).

In the framework of irreversible LSD1 inhibitors that have not yet been tested in patients, INCB059872 (Incyte) and T3775440 (Takeda) are noteworthy. The recently developed INCB059872 is a potent and selective LDS1 inhibitor that delays cellular proliferation and induces differentiation in AML cell lines and primary human AML cells. Moreover, oral administration of INCB059872 significantly inhibited tumor growth and prolonged survival of MLL–AF9 expressing leukemic mice (15). Furthermore, preclinical studies combining INCB059872 with the pro-differentiating agent ATRA showed synergism in promoting AML cells differentiation and apoptosis in human AML cells. Collectively, these data provide a scientific rationale for the clinical evaluation of INCB059872 and ATRA in AML patients (15).

In 2017, Ishikawa et al. described the anti-leukemic activities of another selective and potent LSD1 inhibitor: T3775440. This compound showed selectivity against erythroid and megakaryocytic leukemic cell lines, and promoted growth inhibition and transdifferentiation into granulomonocytic-like cells (68). Mechanistically, the effect of T3775440 was linked to its role in the disruption of LSD1–GFI1B interaction, which is a critical negative regulator in definitive erythroid and megakaryocytic differentiation. As a consequence, T3775440-mediated dissociation of the LSD1–GFI1B complex induces transcriptional derepression of GFI1B target genes leading to cell transdifferentiation and growth inhibition and/or apoptosis in GFI1B-expressing acute erythroid leukemia and acute megakaryoblastic leukemia cell lines (68), thus suggesting its use for the treatment of M6 and M7 AML patients.

### Reversible LSD1 Inhibitors

In recent years, a number of non-covalent reversible LSD1 inhibitors have been generated with the advantages to show a safer metabolic profile, promising preclinical anti-leukemic proprieties, and low nanomolar potency (15). Among these compounds, SP2509 (Salarius Pharmaceuticals) is particularly interesting. Experiments on AML cell lines and AML primary cells demonstrated that SP2509 inhibits cell proliferation and promotes cell differentiation and apoptosis without inducing any un-related *in vivo* toxicity. Interestingly, this compound is lethal against AML expressing MLL-rearrangements and, interestingly, also NMP1 mutation (64), suggesting that use of this compound may be extended beyond the spectrum of MLL-rearranged AMLs.

Overall, these studies indicate that activity of LSD1 inhibitors as single agents may be limited in AML, by being restricted to specific categories of patients. However, rational drug combination studies have shown more promising results in AML clinical trials. For example SP2509 has shown a significant synergistic lethality of primary AML blasts and prolonged survival in xenograft AML mouse models when combined with the HDAC inhibitor panobinostat compared to either agent alone (64). Moreover, as stated at the beginning of this paragraph, a number of LSD1 inhibitors are being tested as differentiation-licensing factors in combination with ATRA (Table 1). Finally, recent data showed that combined inhibition of LSD1 and EZH2 acted synergistically against AML by promoting upregulation of H3K4me1/2 and H3K9Ac and downregulation of H3K27me3, and leading to decreased expression of the anti-apoptotic protein Bcl-2. These epigenetic alterations also compromise mitochondrial respiration capacity and glycolytic activity and resulted in ATP depletion, a key event contributing to the potent cytotoxic effect of the drug combination. These findings report a novel therapeutic approach against AML by combining two small molecules that inhibit different histone methylation-modulating proteins with apparently opposite enzyme activities (69).

## Conclusion

Epigenetic modifying drugs broadly influence gene expression and have the potential to correct the dysregulated transcriptome of AML. Within this category, LSD1 inhibitors constitute a promising epigenetic approach to treat AML, and many clinical trials are currently underway worldwide (15, 18). However, despite significant progress in understanding some functional aspects of LSD1 biology, the biochemical mechanisms underlying its involvement in transcriptional activation are not firmly established, and its blockade may be having broader consequences than anticipated. We expect that in the near future a deeper characterization of the molecular functions of LSD1 in leukemia along with the results of these first clinical studies will result in clearer understanding of epigenetic regulatory mechanisms in AML and improved treatment of patients.

## Author Contributions

SM and RB conceived and designed the review. SM, RB, and DM wrote the paper and engaged in active discussion. DM composed the figures. All authors read and approved the final manuscript.

## Conflict of Interest Statement

The authors declare that the research was conducted in the absence of any commercial or financial relationships that could be construed as a potential conflict of interest.
